# Genome-wide association analysis and QTL mapping reveal the genetic control of cadmium accumulation in maize leaf

**DOI:** 10.1186/s12864-017-4395-x

**Published:** 2018-01-25

**Authors:** Xiongwei Zhao, Longxin Luo, Yanhua Cao, Yajuan Liu, Yuhua Li, Wenmei Wu, Yuzhou Lan, Yiwei Jiang, Shibin Gao, Zhiming Zhang, Yaou Shen, Guangtang Pan, Haijian Lin

**Affiliations:** 1 Maize Research Institute, Sichuan Agricultural University, Chengdu, 611130 China; 2 Department of Agronomy, Purdue University, West Lafayette, 47906 USA

**Keywords:** Maize, Cadmium accumulation association analysis, QTL mapping, Allelic variation, Candidate gene

## Abstract

**Background:**

Accumulation of cadmium (Cd) in maize (*Zea mays* L.) poses a significant risk to human health as it is ingested via the food chain. A genome-wide association study (GWAS) was conducted in a population of 269 maize accessions with 43,737 single nucleotide polymorphisms (SNPs) to identify candidate genes and favorable alleles for controlling Cd accumulation in maize.

**Results:**

When grown in contaminated soil, accessions varied significantly in leaf Cd concentration at both the seeding and maturing stages with phenotypic variation and the coefficient of variation all above 48%. The co-localized region between SYN27837 (147,034,650 bp) and SYN36598 (168,551,327 bp) on chromosome 2 was associated with leaf Cd under three soil conditions varying in Cd content in 2015 and 2016. The significant SNP (SYN25051) at position 161,275,547 could explained 27.1% of the phenotype variation. Through QTL mapping using the IBMSyn10 double haploid (DH) population, we validated the existence of a major QTL identified by GWAS; *qLCd2* could explain the 39.8% average phenotype variation across the experiments. Expression of *GRMZM2G175576* encoding a cadmium/zinc-transporting ATPase underlying the QTL was significantly increased in roots, stems and leaves of B73, a low Cd accumulation line in response to Cd stress.

**Conclusions:**

Our findings provide new insights into the genetic control of Cd accumulation and could aid rapid development of maize genotypes with low-Cd accumulation by manipulation of the favorable alleles.

**Electronic supplementary material:**

The online version of this article (doi: 10.1186/s12864-017-4395-x) contains supplementary material, which is available to authorized users.

## Background

Cadmium (Cd) is a heavy metal that is highly toxic to all organisms and is one of the major environmental pollutants. One primary concern is its transfer from crop plants to the human diet. It is concluded that crop plants contribute more than 70% of cadmium intake in humans [1]. Excessive intake may result in damage from cancers of the prostate, lungs, and testes, kidney tubule damages, emphysema, and bone fractures [2]. Maize (*Zea mays* L.) is not only a global crop but also a primary food source for hundreds of millions of people in developing countries. In Asia, up to 50% of the ingested Cd comes from crops and their products, including maize [3]. Because of a serious concern in widespread food safety, research into a better understanding of the mechanisms driving Cd accumulation in crop plants has become increasingly important.

The pathway of Cd transport has been elucidated in rice by genetic and genomic approaches [4]. There are three main transport processes most likely to mediate Cd accumulation in plants. First, roots take up Cd from soil through the symplastic pathway. In plants, many of the transporters for divalent transition metals have Cd uptake activity. For example, *OsIRT1* controls Cd absorption and overexpression of *OsIRT1* increases Cd accumulation [5]. Secondly, Cd accumulation happens through root-to-shoot translocation via xylem loading. The ability of xylem-mediated Cd translocation into shoots has shown to be a major determinant for shoot Cd accumulation in many plants [6, 7]. *OsHMA3* has been identified as a regulator for xylem Cd transport in rice by mediating vacuolar sequestration of Cd in root cells [8]. Third, Cd is redirected to phloem at nodes and remobilized from leaves to grains. Remobilization of Cd from leaf blades to grains appears to be regulated by phloem transport [9]. In rice, *OsLCT1*in leaf blades plays a role in translocation of Cd from enlarged vascular bundles to diffuse vascular bundles, which connects to the panicle [10]. *OsHMA2* is involved in the preferential distribution of Zn and Cd at the roots and nodes [11]. All research reports suggest that the various transport systems are involved in Cd accumulation and differ in their roles in controlling Cd uptake and translocation.

Cd accumulation is a complex quantitative trait, controlled by multiple genes. Although the mechanisms of Cd accumulation are widespread and comprehensive in rice and *Arabidopsis thaliana*, none of homologous genes that have been involved in the natural genetic variation of Cd accumulation in rice and *A. thaliana* were cloned in maize. Traditionally, quantitative trait loci (QTLs) have been identified using linkage mapping populations such as recombinant inbred lines (RILs) and double haploid lines (DHs). However, such mapping populations are often generated from a cross between two parental lines. As a result, only a limited amount of natural allelic diversity can be captured in the population, leading to the identified QTLs spanning relatively large genomic regions and making identification of causal genes more difficult. Genome-wide association study (GWAS) utilizes the natural populations for QTL detection though marker-trait association [12, 13]. Integration of linkage mapping and GWAS provides a more powerful tool for identifying and verifying candidate genes underlying complex traits [14]. To date, genetic control of Cd accumulation is not well understood in crop species, particularly for marker and gene identification using integrated approaches of linkage mapping and GWAS. In this study, we assessed Cd accumulation in 269 maize genotypes grown at different levels of Cd contaminated soils in a greenhouse and under field conditions and conducted GWAS for leaf Cd concentration with the Illumina Infinium maize SNP50K. In addition, a co-located region identified by GWAS was validated through linkage mapping in a ten-generation intermated B73 × Mo17 (IBMSyn10) DH population with an ultra-high density bin map. Candidate genes and favorable alleles identified in the diversity accessions would assist in further revealing genetic control of Cd accumulation in maize.

## Results

### Phenotypic variation and heritability

Two hundred and sixty nine diverse accessions were evaluated for Cd accumulation of leaves under low-Cd (LSLCd) and middle-Cd (MSLCd) conditions at the seeding stage and under high-Cd condition at the maturing stage (HLCd) in 2015 and 2016, respectively. Analysis of variance (ANOVA) indicated that diverse accessions differed significantly (*P* < 0.01) in Cd concentration in leaves, while significant genotype by environment (Y × G) interactions were observed (*P* < 0.01) (Table 1). The cultivation environment had a great impact on Cd accumulation.

**Table 1:** Phenotypic variations for leaf Cd concentration in 269 maize accessions in experiments conducted in 2015 and 2016

Year	Trait	No.	Mean ± SD^a^ (mg·kg^−1^)	Rang (mg·kg^−1^)	CV (%)^b^	F_Y_^c^	F_G_^d^	F_G × Y_^e^	*h* ^*2*f^
2015	LSLCd	251	1.73 ± 1.28	0.14–5.90	73.8	90.6^**^	2.07^**^	0.24	0.82
	MSLCd	238	25.9 ± 13.8	3.22–64.3	53.6	239.9^**^	3.53^**^	1.27^**^	0.71
	HLCd	250	47.3 ± 23.0	16.6–128.4	48.7	1178.2^**^	9.50^**^	1.64^**^	0.62
2016	LSLCd	269	1.52 ± 1.28	0.18–5.36	84.2				
	MSLCd	249	18.6 ± 9.7	2.79–49.6	52.9				
	HLCd	243	21.9 ± 13.9	6.76–83.3	63.6				

The F-test was applied to determine the significance level

** indicates significance at level of 0.01

^a^ SD standard deviation

^b^ variation coefficient (CV)

^c^ year

^d^ genotype

^e^ genotype × year

^f^ Broad-sense heritability (*h*^*2*^)

**Fig. 1:**
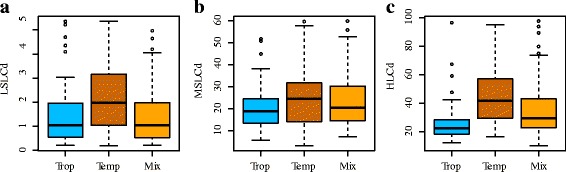
Distribution of subgroups in maize lines under low-Cd condition (**a**) and middle-Cd condition (**b**) at seeding stage and under high-Cd field condition at maturing stage (**c**). Trop = Tropical group; Temp = Temperature group; Mix = other group with no clear identity

The mean HLCd value for individual accessions ranged from 6.76 to 128.4 mg·kg^−1^ under the high-Cd field condition at the maturing stage. Higher values for HLCd were observed in 2015 with a mean of 47.3 mg·kg^−1^, compared with 2016 (mean = 21.9 mg·kg^−1^) (Table 1). At the seeding stage, the mean Cd concentration of leaves (LSLCd) at the low-Cd level ranged from 0.14 to 5.90 with a mean of 1.61 mg·kg^−1^, whereas the Cd concentration of leaves at the middle-Cd level (MSLCd) varied from 2.79 to 64.3 mg·kg^−1^, with an average of 22.3 mg·kg^−1^. The results showed that the Cd accumulation of leaves in the natural population had a large variation with variation coefficient (CV) over 48%. In addition, the mean value of leaf Cd concentration in the tropical group was significantly lower than that in the temperate group (*P* < 0.001) in different environments (Fig. 1). Furthermore, the broad-sense heritability (*h*^*2*^) for Cd accumulation across all measured environments ranged from 0.62 (HLCd) to 0.82 (LSLCd), indicating high repeatability over testing environments as well as roles of genetic factors in determining the trait. Overall, the maize plants exhibited significant genetic variations in Cd concentration when grown at different Cd levels.

### Population structure and linkage disequilibrium (LD)

**Fig. 2:**
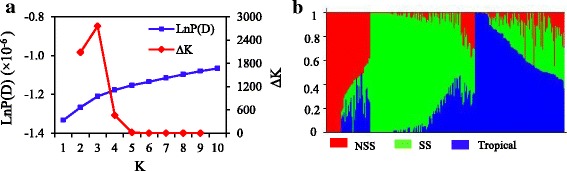
Analysis of the population structure of 269 maize inbred lines estimated from 5200 SNPs. **a** Estimated LnP(D) and *∆k* over five repeats of STRUCTURE analysis; **b** Population structure of the 269 lines from K = 3. SS = Stiff Stalk; NSS = Non-Stiff Stalk

**Fig. 3:**
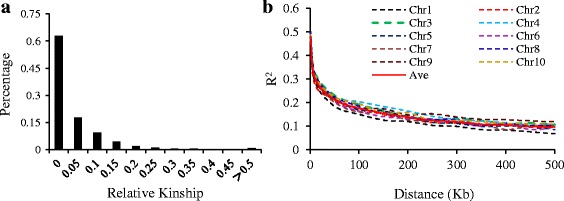
The distributions of pair-wise kinship and LD decay rate per chromosome. **a** The distributions of pair-wise kinship between 269 inbred lines; **b** LD decay rate per chromosome based on mean r^2^ per 50 kb region

The population structure was calculated using 5200 SNPs. Because the log likelihood of data (LnP(D)) from the STRUCTURE output continuously increased with K value, it was not capable of identifying groups. Accordingly, the ∆K value was calculated for each K, suggesting that the 269 genotypes could be assigned into three groups (Fig. 2a). The accessions were divided into tropical, stiff stalk (SS) and non-stiff stalk (NSS) groups. Among them, 50 individuals were assigned to the NSS group, 101 individuals to the SS group, and 118 individuals to the tropical group (Additional file 1). Among them, 92 lines had mixed assignment into these three groups. The average relative kinship between any two lines was 0.067. A total of 62.8% of kinship coefficients were 0 while 27.2% were between 0 and 0.2 (Fig. 3a), indicating a weak relative kinship in the diverse population.

The extent of LD was estimated with all 43,737 SNPs using TASSEL 5.0. A rapid decline in LD was observed with increasing physical distance on all chromosomes (Fig. 3b), but the decay rate varied over different chromosomes. At a cutoff of r^2^ = 0.1, equilibrium was reached within 200–250 kb on chromosome 1, 250–300 kb on chromosome 2, and 300–600 kb on the rest of the chromosomes. The mean LD decay was 350–400 kb across all chromosomes.

### Genome-wide association analysis of Cd accumulation

To determine which model was more suitable for association mapping analysis, we used and compared four models for testing each trait. QQ plots showed that the distribution of observed -log_10_(*P*) values from the simple model and Q model departed far from the expected distribution, leading to a high level of false-positive signals. The model controlling K and Q + K had similar effects on reducing the false positives, except that LSLCd16 in the Q + K model had lower power than the K model (Additional file 2). Thus, we selected the Q + K model to identify association signals for LSLCd15, MSLCd15, HLCd15, MSLCd16, HLCd16, and the Q model for LSLCd16.

**Fig. 4:**
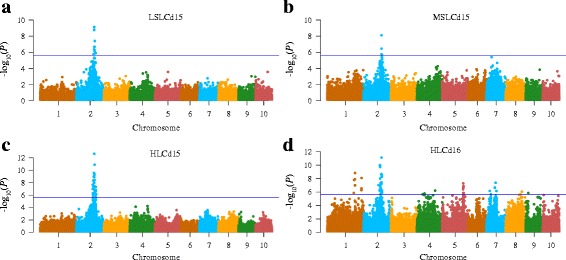
Manhattan plots of association analysis between leaf Cd concentration and single-nucleotide polymorphism (SNP) markers in maize. The horizontal dashed blue line represents the significance threshold -log_10_(*P*) = 5.94. Genome-wide association (GWA) mapping under low-Cd condition (**a**) and middle-Cd condition (**b**) at the seeding stage in 2015. GWA mapping of leaf Cd concentration under high-Cd field condition at maturing stage of maize in 2015 (**c**) and 2016 (**d**)

Across three soil conditions, 63 SNPs located on 5 of the 10 chromosomes were significantly associated with leaf Cd concentration (*P* < 1.18 × 10^−6^) (Fig. 4, Additional file 3). The association explained approximately 15.9% of the phenotypic variations. The greatest number of significant SNPs were found with traits collected from the high-Cd field environment (HLCd16, 47 SNPs) at the maturing stage in 2016, followed by the same condition (HLCd15, 30 SNPs) in 2015. However, no significant associations were detected between SNPs and leaf Cd concentration under the low-Cd condition in 2016 (Additional file 4).

Forty SNPs in a single region on chromosome 2 were highly associated with leaf Cd concentration (Fig. 4, Additional file 3). Meanwhile, the co-localized peak between SYN27837 (147,034,650 bp) and SYN36598 (168,551,327 bp) explained an average of 17.5% of the phenotypic variation, based on R^2^ values. SYN25051 (A/G), the most highly significant SNP with -log_10_(*P*) = 13.7 at position 161,275,547 on Chr2, explained 27.1% of the phenotype variation. In addition, 5 SNPs located on chromosome 5 and 6 SNPs located on chromosome 7 were associated with HLCd16 with R^2^ values ranging from 12.3 to 17.3%. Nine SNPs on chromosome 1were significantly associated with HLCd16.

### QTL mapping for Cd accumulation in IBM Syn10 DH population

An ultra-high density bin map was used to further confirm the QTL identified from GWAS. The IBMSyn10 DH population was created by crossing the high leaf Cd line of Mo17 with an A at Chr2:161,275,547 with a low leaf Cd line of B73 with a G at Chr2:161,275,547 (Additional file 5). The field results showed that the leaf Cd concentration in B73 (23.3 mg·kg^−1^) was significantly lower than that in Mo17 (64.1 mg·kg^−1^). Leaf Cd concentration ranged from 9.5 to 114.2 mg·kg^−1^ in the mapping population, but the phenotypic distribution exhibited a transgressive segregation (Additional file 6). The variation in leaf Cd concentration suggested that Cd accumulation was controlled possibly by major-effect QTLs.

**Fig. 5:**
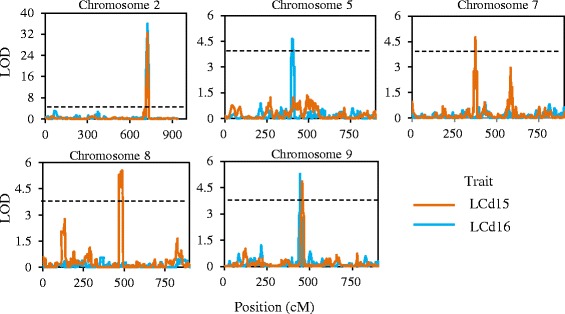
Logarithm of odds (LOD) score curves for the QTLs for the leaf Cd accumulation of IBMSyn10 DH population in 2015 and 2016. Gray dotted line indicates LOD threshold (3.84)

Composite interval mapping was performed using the threshold of LOD ≥ 3.84. Five QTLs for leaf Cd concentration were mapped and located on chromosomes 2, 5, 7, 8, and 9 (Table 2, Fig. 5). Noteworthy, an overlapped major QTL named qLCd2 (a QTL for leaf Cd concentration on chromosome 2 from GWAS) was identified, spanned a 13.83 Mb region (153.75–167.58 Mb) and explained 41.2% and 38.4% of the phenotypic variation in 2015 and 2016, respectively. This major QTL had positive additive effects, which indicated that B73 contributed more to Mo17. Although LOD values of the other 4 QTLs were high, those QTLs explained only 3.4% to 4.4% of the phenotypic variation for leaf Cd accumulation (Table 2). No QTLs in other chromosomal regions were observed by explaining more than 5% of the variation in leaf Cd accumulation, suggesting that the qLCd2 is the major genetic locus controlling natural variation in leaf Cd accumulation when maize is grown in Cd contaminated soil.

**Table 2:** QTLs detected for leaf Cd accumulation in maize by composite interval mapping

No.	Year	QTL	Chr	Pos (cM)	LOD^a^	R^2^ (%)	A	Support region (cM)^b^	Physical distance (Mb)^c^
1	2015	qLCd2	2	723.65	32.42	41.24	24.22	714.99–728.64	154.02–164.30
	2016			723.01	36.04	38.41	13.25	712.26–730.91	153.76–167.58
2	2016	qLCd5	5	401.08	4.66	3.36	3.91	394.16–415.08	21.30–29.05
3	2015	qLCd7	7	374.39	4.76	3.75	5.97	370.29–378.47	113.68–119.73
4	2015	qLCd8	8	488.31	5.56	4.44	−5.62	468.15–491.48	103.10–105.60
5	2015	qLCd9	9	456.11	4.86	3.84	5.27	447.91–462.47	95.85–98.82
	2016			444.46	5.27	4.29	6.92	442.45–445.18	93.45–95.08

*Chr* chromosome, *Pos* position of peak with highest logarithm of odds(LOD), *R*^*2*^ explained phenotypic variance, *A* additive effect of B73 allele

^a^ after 1000-permutation tests, threshold values of LOD for Cd concentration of leaves was calculated as 3.84

^b^ the position of support regions which was determined by half LOD at the peak in cM

^c^ the physical distance (Mb) of the bin makers corresponding to genetic distance

### Prediction of candidate genes

**Fig. 6:**
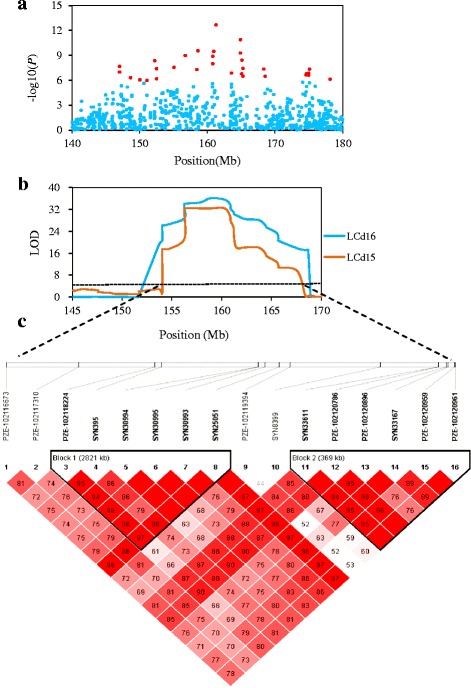
Genome-wide association analysis detected a significant signal associated with leaf Cd concentration in maize. **a** Manhattan plot of association analysis for HLCd15; **b** The QTL associated with leaf Cd accumulation in maize at chromosome 2. **c** Linkage disequilibrium (LD) between the SNPs in target region (Chr2: 153.75–167.58 Mb) and the magnitude of LD indexed by the D’ statistic. Red squares without numbers indicate complete LD (D’ = 1, P < 0.01). D’ values are shown in the squares for values < 1.0

Based on the results of GWAS and QTL mapping, a major and co-located QTL was associated on chromosome 2, which encompassed a 13.83 Mb region (153.75–167.58 Mb) (Fig. 6a, b). In the comparatively narrow region, GWAS detected a highly significant cluster of 16 SNPs. To determine the trait-associated loci, all significant SNPs located in the target region were clumped at D’ > 0.80. We observed that these 12 SNPs exhibited strong LD and could form two LD blocks (Fig. 6c), which caused an overlap with QTL mapping. The first LD block spanned around 3 Mb including 6 SNPs and the second big LD block spanned around 400 kb including 6 SNPs. Candidate genes were predicted based on the 12 SNPs and their extension regions from 300 kb upstream to 300 kb downstream (LD distance of chromosome 2).

**Fig. 7:**
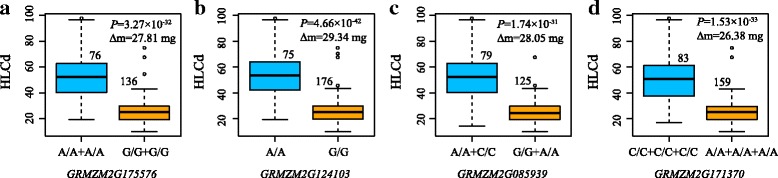
The boxplot of phenotype analysis between the candidate genes for locus associated with leaf Cd concentration in maize and phenotypic difference between minor alleles and major alleles. The number above box represents the number of inbred lines homozygous for a determined allelic variant. Δ*m*, the difference of mean of leaf Cd concentration between the minor alleles and major alleles at maturing stage over 2 years and 2 replications. **a** Two combinations of the minor and major between SYN395 and SYN30994 in *GRMZM2G175576*and *GRMZM2G455491*region; **b** The single SYN25051 loci in *GRMZM2G124103*; **c** Two combinations of the minor and major between SYN33611 and PZE-102120786 in *GRMZM2G085939*region; **d** Three combinations of the minor and major among SYN30994, SYN30995 and SYN30993 in *GRMZM2G171370* region

Detailed descriptions of 50 candidate genes were summarized in Additional file 7. According to the gene functional annotations in the maize B73 genome (RefGen_v2), 8 causal candidate genes were predicted for 12 loci associated with Cd accumulation (Table 3). Among these genes in the first LD block, *GRMZM2G455491* and *GRMZM2G175576*, simultaneously encoding a cadmium/zinc-transporting ATPase, were located at 64.18 kb and 42.64 kb upstream of the PZE-102118224 and SYN395 loci, respectively. On average, the individuals carrying the major frequency alleles (G/G + G/G) of two SNPs (PZE-102118224, SYN395) had 27.8 mg lower leaf Cd content than those with minor frequency alleles (A/A + A/A) (Fig. 7a). A putative gene, *GRMZM2G124103*, encoding a vacuolar ATPase, was found at 72.3 kb apart from the peak SNP (SYN25051). The individuals carrying the major frequency allele (G/G) had 29.3 mg Cd content higher than those with the minor frequency allele (A/A) (Fig. 7b). A ZIP transcription factor, encoded by the putative gene *GRMZM2G171370*, was targeted by three SNPs, SYN30994, SYN30995, SYN30993. On average, there was 26.4 mg difference in leaf Cd content between major and minor alleles in three SNPs (Fig. 7d). In the second LD block, SYN33611 and PZE-102120786 were located 85.30 kb upstream and 81.87 kb downstream of *GRMZM2G085939*, respectively. The leaf Cd concentration of individuals with minor frequency alleles (G/G + A/A) at these loci was 28.1 mg higher than those with major frequency alleles (A/A + C/C) (Fig. 7c).

**Table 3:** Significant associations and corresponding QTLs detected by genome-wide association study and QTL mapping of leaf Cd concentration at seeding and maturing stage in maize

No.	Possible causative SNP	Allele^a^	MAF	Position (bp)	-log_10_(*P*)	R^2^ (%)	Candidate gene	Function description	Distance to Gene^b^
1	PZE-102118224	A/G	0.397	158,453,594	7.29	15.25	GRMZM2G455491	Cadmium/zinc-transporting ATPase	64.18 kb
							GRMZM2G175576	Cadmium/zinc-transporting ATPase	42.64
2	SYN395	A/G	0.376	158,609,741	9.54	20.1			202.75 kb
3	SYN30994	A/C	0.387	160,798,026	7.99	16.74	GRMZM2G171370	ZIP transcription factor	158.95 kb
4	SYN30995	A/C	0.378	160,798,113	8.89	18.67			158.86 kb
5	SYN30993	A/C	0.352	160,957,319	9.48	23.03			Intron
6	SYN25051	A/G	0.308	161,275,547	12.7	27.12	GRMZM2G124103	Vacuolar ATPase G subunit	72.26 kb
7	SYN33611	A/G	0.386	164,889,910	9.28	19.52	GRMZM2G085939	Calmodulin-binding heat-shock protein	85.30 kb
8	PZE-102120786	A/C	0.329	164,893,346	10.9	23.1			81.87 kb
9	PZE-102120896	A/G	0.416	165,068,962	6.77	14.16	GRMZM2G386138	Cupin domain containing protein	Intron
10	SYN33167	A/G	0.366	165,105,090	8.42	17.66	GRMZM2G047727	Ubiquitin fusion protein	Intron
11	PZE-102120959	A/G	0.414	165,259,741	6.49	13.56	GRMZM2G085153	Nucleic acid binding protein	2.67 kb
12	PZE-102120961	A/C	0.426	165,259,881	7.43	15.54			2.53 kb

^a^ The letter under the line is the nucleotide minor frequency

^b^ Genes identified within 300 kb of SNPs detected by the distant of LD decay are indicated

### Expression analysis of candidate genes

**Fig. 8:**
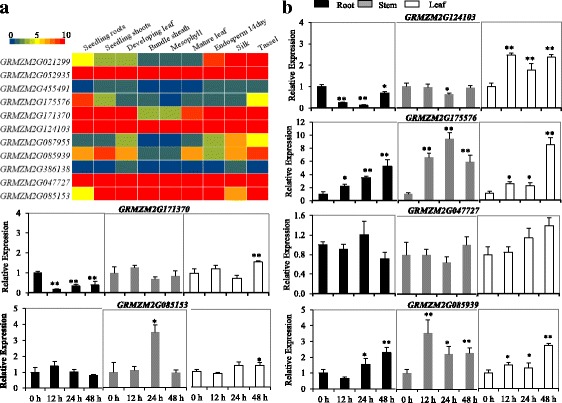
Expression profiles of putative genes. **a** A heat map illustrating levels of gene expression of the putative genes in nine different tissues from various developmental stages. **b** Relative levels of gene expression in maize B73 roots, stems and leaves for response to Cd stress; Cd (200 mg/L) treat for 0 h (control), 12 h, 24 h, 48 h samples

Expression profiles of eight candidate genes were examined in maize B73 under non-limiting growth conditions. Using online transcriptome data from maize B73 (MaizeGDB), an expression heatmap was constructed for these candidate genes in different tissues from 8 developmental stages (Fig. 8a). The results showed that the expression patterns of different candidate genes varied greatly in different tissues. *GRMZM2G124103* and *GRMZM2G047727* showed a relatively high level of expression in all developmental stages compared to the other candidate genes. *GRMZM2G175576* in roots had a relatively high level of expression compared to other tissues. On the contrary, *GRMZM2G455491* and *GRMZM2G386138* exhibited a constitutively low level of expression in different tissues. The expression of 6 candidate genes were examined in leaves, stems and roots of Cd-stressed B73 seedlings by quantitative real-time PCR analyses. As illustrated in Fig. 8b, a dramatic upregulation of the *GRMZM2G175576* gene was observed in response to Cd stress, especially in the stems, exhibiting about a 9.5-fold increase in transcript abundance compared to the control. The relative expression levels of *GRMZM2G085939* also significantly increased in the stems and leaves in response to Cd stress. In contrast to the other candidate genes, expression levels of *GRMZM2G124103* and *GRMZM2G171370* significantly decreased in roots and stems under Cd stress. The expression of *GRMZM2G047727* and *GRMZM2G085153* had minor response to Cd exposure. These results suggested that 4 candidate genes possibly played diverse roles in maize development and Cd-stress response and tolerance. Furthermore, possible sub-cellular locations of the candidate genes were predicted using ProtComp 9.0. This prediction suggested that *GRMZM2G175576, GRMZM2G124103* and *GRMZM2G171370* might be localized in plasma membrane, and vacuolar and nucleus organelles, respectively (Additional file 8).

## Discussion

Diverse responses of maize accessions to Cd treatments provided valuable information about the range and distribution of Cd concentration in maize and offered new insights into germplasm enhancement for low Cd accumulation. The mean leaf Cd concentration in the temperate group was nearly double (*P* = 8 × 10^−13^) that in the tropical group under high-Cd condition (Fig. 1). The relative values of Cd concentration in the three subpopulations (tropic < Mix < SS < NSS) were consistent with the levels of genetic relatedness of these associations to biotic and abiotic stresses [15, 16]. The results suggested that the tropical germplasm contained alleles that would provide useful information for exploring genetic variations in reducing Cd accumulation within SS and NSS. The low-Cd level in the eight lines of 11GP66-1, T32, 98WV9, Wa138, CIMMYT-1, B047, Y8G and CML282 indicated that these lines could be used for developing new maize varieties with low-Cd accumulation.

Cd accumulation is a complex quantitative trait, controlled by multiple genes. Most previous research on the mechanisms of Cd accumulation has focused on rice and *A. thaliana*. In rice, a major QTL controlling the translocation of Cd from roots to shoots at the seedling stage was mapped [17]. Through GWAS, Chao et al. successfully identified a single strong peak of SNPs associated with leaf Cd accumulation in *A. thaliana* [18]. In this study, we found 63 loci associated with leaf Cd accumulation through GWAS. Noteworthy, we observed a single region on chromosome 2 that contained 40 SNPs highly associated with leaf Cd concentration (Fig. 2). Across all experiments in 2015 and 2016, a major locus identified for Cd accumulation through GWAS was successfully validated through QTL mapping (Fig. 5). This QTL could explained over 38% of the phenotypic variation (Table 2), implying that Cd accumulation in leaves could be controlled by a major QTL. The remaining minor novel QTLs are also of interest but further work is needed to validate these QTLs in controlling Cd accumulation.

In the comparatively narrow region, GWA detected a highly significant cluster of 16 SNPs on chromosome 2, which extended to the candidate region to a 300 kb window. According to their putative functions, the candidate genes in support region are primarily involved with DNA binding, catalytic activity, transcription regulator activity and transport. Within the QTL support intervals, the R^2^ of all markers were over 13.6%, indicating that all these markers had high LD with the causal gene. According to a previous study, LD has been observed for the chromosomes that could be involved in the domestication process and selection signatures [19]. More importantly, a rapid breakdown of linkage-related LD can be favorable for association testing of candidate genes that are located nearby the mapped QTL and have functional relevance to trait variation [20]. Among these loci on the target region, 12 potential causative loci exhibited the strongest associations and rapid LD (Fig. 6c). Therefore, the LD might cause overlapping effects and/or co-selection by reorganization, mutation, and selection in the evolution process. These chromosome segments can be incorporated into breeding efforts in cultivating a low-Cd accumulation germplasm.

Knowing genes within a QTL region can help understand trait architecture if their function can be related to the associated trait. Once metal ions are absorbed, translocation of Cd from the roots to the shoots requires loading of Cd into the xylem from the symplast in the stele. Xylem loading of Cd in plants requires Heavy Metal ATPases [4]. In this study, *GRMZM2G175576*, close to the SNP of PZE-102118224, was predicted to encode a heavy metal transporting ATPase, which was homologous to rice clone-gene *OsHMA3* [21]. *OsHMA3* has been identified as a firewall by sequestrating Cd into the vacuoles in the roots, keeping the Cd away from the above-ground tissues [8, 22]. OsHMA3 is localized to the vacuolar membrane, but GRMZM2G175576 is predicted to be localized to the plasma-membrane (Additional file 8). The plant plasma membrane is regarded as the first ‘living’ structure that is a target for heavy metal toxicity [23]. These plasma membrane proteins, such as OsHMA2, also mediate Cd transport in plants. Thus a high expression of such proteins might reduce Cd accumulation in the plant [24]. Exposure of plants to Cd in nutrient solution for 12 h caused significant increase in the expression levels of *GRMZM2G175576* in roots, stems and leaves of B73 (Fig. 8). All these results indicate that the putative protein *GRMZM2G175576*, played an important role in controlling Cd accumulation of maize.

Excess toxic metal may activate defense mechanisms against these toxic metals [25]. Among all defense mechanisms, vacuolar sequestration and plant-mediated bioremediation have received attention [26]. We found that *GRMZM2G124103* encoding a vacuolar-type ATPase was located 72.3 kb apart from the peak SNP (SYN25051), sharing 77% amino acid sequence identity with the *AVMA10*. V-ATPase, a type of H pump, could maintain or adjust the concentration balance of cations on both sides of a membrane, including Na^+^, Ca^2+^, and Cd^2+^ [27]. In a previous report, *LbVHA-c1* may confer stress tolerance through tolerance peroxidase and superoxide dismutase activities, protecting membranes from damage and decreasing lipid peroxidation under salt stress [28]. These protein pumps control the movement of ions across the vacuolar membrane, possibly leading to increased Cd tolerance of the plant. *GRMZM2G085939*, located downstream of PZE-102120786, encodes a calmodulin binding heat shock protein. Heat shock proteins are expressed in organisms at temperatures above the optimum growth temperatures. HSP17 and HSP70 have been involved in root responses of peruvian tomato (*Lycopersicon peruvianum*) to Cu and Cd [23]. We found that the expression of *GRMZM2G085939* significantly increased in roots, stems, and leaves in response to Cd stress, suggesting that *GRMZM2G085939* might be involved in protection and repair of the cellular proteins.

## Conclusions

In briefly, temperate varieties of maize had twice the Cd concentration as tropical varieties. One region identified by GWAS was co-localized with quantitative trait loci (QTL) and linkage mapping. Three candidate genes, *GRMZM2G175576, GRMZM2G124103,* and *GRMZM2G085939,*underlying the major QTL were proposed, including a gene (*GRMZM2G175576*) homologous to rice genes (*OsHMA3*, *OsHMA2*) that functioned similarly in phenotypic response. Future work will include cloning the genes and illustrating the molecular mechanisms for controlling Cd accumulation in maize plants. Meanwhile, the identified QTL region could be utilized for the development of Cd resistant plants.

## Methods

### Plant materials

Two hundred and sixty nine maize accessions for the GWAS were selected from the Southwest China breeding program [29], including 17 parents of major expanded hybrids from Southwest China, 160 newly improved inbred lines, 35 representative inbred lines of temperate germplasm (Reid, Lancaster, etc.), and 62 CIMMYT and US imported lines and tropical germplasm. Detailed information of the accessions is listed in Additional file 9. Meanwhile, an IBMSyn10 DH population [30], including 197 doubled haploid lines, was used for QTL analysis.

### Plant growing conditions

Two experiments were conducted for GWAS in maize. The seeding experiment was conducted in pots in a green house at Sichuan Agricultural University (Yaan, Sichuan, China; N 30^o^08’, E 103^o^14’) during the months of October, 2015 and April, 2016. Eight seeds of each of 269 accessions were initially sown in plastic pots (22 cm diameter and 28 cm deep) containing 14 kg clayey soil with low Cd and pH of 6.3, and three seedlings were kept after germination. Plants were grown in the greenhouse for 12 d prior to Cd treatment. Cd treatments were applied as Cd solutions with CdCl_2_·2.5H_2_O at concentrations of 0 (low-Cd level, available Cd of 3.28 mg·kg^−1^ in soil) and 0.1 mmol·kg^−1^ (middle-Cd level, available Cd 18.8 mg·kg^−1^ in soil). The average air temperatures in the greenhouse were 29 °C/21 °C (day/night). Irrigation was controlled manually during the experiment to avoid loss of mineral elements and Cd caused by excessive water. After 15 d of growth, the third and fourth leaves were harvested for measuring Cd concentration. The greenhouse experiment was a randomized complete block design with two replications.

A field trial for GWAS was conducted in the summers of 2015 and 2016 in Deyang city, Sichuan province of China (104°06’N, 31°11′E). Initially, 269 accessions were grown in a greenhouse for 2 weeks under environmental conditions described above. To ensure and obtain uniform plant growth, seedlings were then visually selected and transplanted to the field for phenotypic trait evaluation. Plants were grown in the contaminated soil with 32.5 mg·kg^−1^ of Cd (high-Cd level). Each 3 m row contained 14 plants 0.80 m apart. The maintenance of plants in the field followed routine practices of maize during the experiment. After seeds matured, five consecutive plants were chosen from the middle of each row, and the middle leaf below the tassel was harvested for measuring Cd concentration.

For the QTL mapping study, plants of IBMsyn10 DH population were initially grown in a greenhouse for 2 weeks and transplanted to the field. Phenotypic traits were collected from the matured plants of IBMsyn10 DH population in the summers of 2015 and 2016 in Deyang city, Sichuan province of China (104°06’N, 31°11′E). Plant growing conditions and the procedures of plant sampling were the same as described previously. The field experiment followed a complete randomized plot design with two replications for both GWAS and QTL mapping for both years.

### Determination of Cd concentration and phenotypic data analysis

All samples were washed thoroughly with tap water to remove soil and dirt, and then were washed with deionized water three times. The leaves were dried and ground until 95%of the sample could pass through a 1 mm screen. The powdered sample (0.5 g) was digested with 20 mL of 67% concentrated nitric acid (HNO_3_) and 33% hydrogen-peroxide (H_2_O_2_) on a heating block at 90 °C for 1 h and 180 °C for 5 h. The digested solutions were filtered after dilution with deionized water. Subsequently, the Cd concentration in the solutions was measured by using the inductively coupled plasma-atomic emission spectrometry (ICP-MS) (Nippon-Jarrell-Ash, Tokyo, Japan).

Analysis of variance (ANOVA) and heritability (*h*^2^) of Cd concentration in leaves were performed using SPSS statistics 21.0. Broad-sense (*h*) was estimated as *h*^2^ = σ_g_^2^/(σ_g_^2^ + σ_ge_^2^ + σ_e_^2^), where σ_g_^2^, σ_e_^2^, σ_ge_^2^ represent TypeIIISS (sums of squares) for genotype (G), environment (E), and interaction variance of G and E, respectively [31, 32].

### Population structure, relative kinship and linkage disequilibrium analysis

In this study, the maizeSNP50K was used to genotype the population as described by Zhang et al. [29]. Through removal of missing rate > 20%, heterozygosity >20% and minor allele frequency (MAF) < 0.05, a total of 43,737 high-quality SNPs were obtained for association analysis.

Population structure (Q matrix) of 269 maize individuals was estimated from a randomly selected set of 5200 high-quality SNPs using STRUCTURE 2.3.4 software with the “admixture model” [33]. The parameter settings for estimating membership coefficients for lines in each subpopulation consisted of a burn-in length of 50,000 followed by 50,000 iterations for each of the clusters (K) from 1 to 10, with each K being run five times. Maximum likelihood and delta K (∆K) tests were used to determine the optimum number of subgroups. The relative kinship (K matrix) between lines was calculated using SPAGeD software [34]. All negative values from this software were set to 0.

Linkage disequilibrium (LD) between pairs of SNPs was estimated by using squared allele frequency correlations between two loci (r^2^) in TASSEL 5.0 [35]. Standardized disequilibrium coefficient (D’) and common haplotype patterns were assessed in Haploview version 4.2 [36]. Haplotype blocks were defined with the confidence interval method [37].

### Genome-wide association analysis

Marker-trait association analysis was performed by TASSEL 5.0 with the General Linear Model (GLM) and Mixed Linear Model (MLM) procedures [35] to control for population structure (Q) and relative kinship (K). The simple linear model (S), Q (Q matrix) model, K (K matrix) model, and Q + K model were applied to assess the suitability of each model for false-positive correction using quantile-quantile (QQ) plots for association analyses. QQ plots and Manhattan plots were generated using the ‘qqman’ package in R. Associations between SNPs and traits were considered significant only if the *P*-value was lower than threshold *P*_threshold_ = 0.05/N, where N was the total number of SNP markers.

### QTL mapping

Genotypic data previously obtained for the IBMsyn10 DH population was used for this QTL mapping [38]. The linkage map included 5955 bins with an average bin size of 344 kb. QTL for leaf Cd accumulation was detected using the composite interval mapping method and Model 6 of the Zmapqtl module of QTL Cartographer 1.17 [39]. The LOD threshold was determined by 1000 permutations and a 10 cM window with scanning intervals at 1 cM interval. Signals were treated as separate QTLs when their peaks were more than 20 cM apart. The support interval of a QTL was defined as the segment of the chromosome in which the LOD at the peak decreased by half [40].

### Prediction of candidate genes and expression analysis

We used the maize B73 reference genome (B73 RefGen_v2, https://www.maizegdb.org/) [41] to identify candidate genes that were either included or close to the significantly associated SNPs. Based on overlapping regions of GWAS and QTL mapping, a region of approximately 300 kb around the SNP was examined for annotated genes putatively involved in iron transporter and/or regulation by transcription factor [4]. To validate the expression levels of candidate genes obtained by GWAS, expression patterns of candidate genes in different tissues of maize under non-limiting growth conditions were analyzed using online data from MaizeGDB. In addition, the responses of each candidate gene to Cd stress were analyzed by quantitative real-time PCR (qRT-PCR). Briefly, the 2-week plants of the B73 line were grown in ½ Hoagland’s nutrient solution amended with CdCl_2_·2.5H_2_O (200 μmol·L^−1^) for 0 h (control), 12, 24, and 48 h. Total RNA was extracted using TRIZOL reagent (Invitrogen, USA) and RNase-free DNase (Takara, Japan) from the roots, stems and leaves. cDNA was synthesized using 1 mg total RNA from each sample by PrimeScript RT Reagent Kit With gDNA Eraser (Perfect Real Time, Takara, Japan). The primer sequences are shown in Additional file 10. Subsequently, qRT-PCR was conducted using the SYBR premix Ex Taq kit (Takara) on an ABI 7500 Real-Time System (Applied Biosystems) and the expression of *GADPH* was used for normalization. Three replicates were used to calculate expression levels of candidate genes by using the 2^-ΔΔCT^ method [42] for each sample. Putative protein sub-cellular localization was predicted using ProtComp version 9 (http://linux1.softberry.com/berry.phtml) which compared homologous proteins of known localization and pentamer distributions in the LocDB and PotLocDB databases.

## Additional files

Additional file 1: Table S1. The population structure analysis for accessions using 5200 SNPs by Structure 2.2.3. (XLS 53 kb)

Additional file 2: Figure S1. Quantile-quantile (QQ) plots for leaf Cd concentration at seeding stage and maturing stage of maize. (PNG 104 kb)

Additional file 3: Table S2. SNPs significantly associated with leaf Cd concentration in maize. (XLS 40 kb)

Additional file 4: Figure S2. Manhattan plots of association analysis for leaf Cd concentration at seeding stage of maize in 2016. (PNG 64 kb)

Additional file 5: Table S3. Haplotype analysis of polymorphic SNPs contained in the overlapped region (153.75–167.58 Mb) on chromosome 2. (XLS 84 kb)

Additional file 6: Figure S3. The frequency distribution of leaf Cd concentration in maize IBMSyn10 double haploid (DH) population. (PNG 7 kb)

Additional file 7: Table S4. Details of the candidate genes in the overlapped region and their putative function. (XLS 30 kb)

Additional file 8: Table S5. The prediction analysis of putative protein sub-cellular localization. (XLS 28 kb)

Additional file 9: Table S6. Pedigree information of 269 maize accessions used in this study. (XLS 45 kb)

Additional file 10: Table S7. Primers of qRT-PCR assay used for quantifying expression levels of candidate genes within a QTL interval. (XLS 26 kb)

## Abbreviations

CIM: Composite interval mapping
CIMMYT: International Maize and Wheat Improvement Center
GLM: Linear model
GWAS: Genome-wide association study
HLCd: The Cd concentration of leaves under the high-Cd condiction at the maturing stage
IBM Syn10 DH population: Intermated B73 × Mo17 synthetic 10 doubled haploid population
K: Relative kinship
LD: Linkage disequilibrium
LOD: Logarithm of odds
LSLCd: Cd concentration of leaves under low-Cd condition at the seeding stage
MLM: Mixed linear model
MSLCd: The Cd concentration of leaves under the middle-Cd condition at seeding stage
nSS: Non-stiff stalk
Q: Population structure
QQ: Quantile-quantile
QTLs: Quantitative trait locus
R^2^: Percentage of phenotypic variation explained by the identified SNPs
SNP: Single nucleotide polymorphisms
SS: Stiff stalk
