# Supplementary figures and images for: Genome-wide association analysis and QTL mapping reveal the genetic control of cadmium accumulation in maize leaf
Source: BMC Genomics. 2018 Jan 25;19:91. doi: 10.1186/s12864-017-4395-x (PMC5785805; doi:10.1186/s12864-017-4395-x)

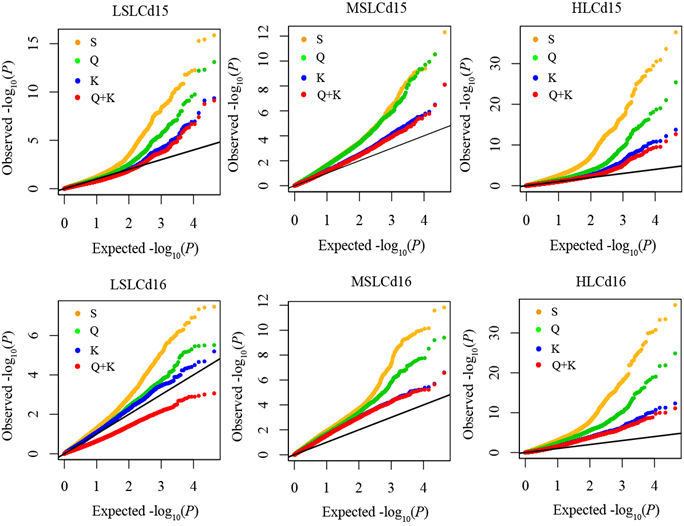

Supplement: Supplementary file 2 — Quantile-quantile (QQ) plots for leaf Cd concentration at seeding stage and maturing stage of maize. (PNG 104 kb) [file 12864_2017_4395_MOESM2_ESM.png]

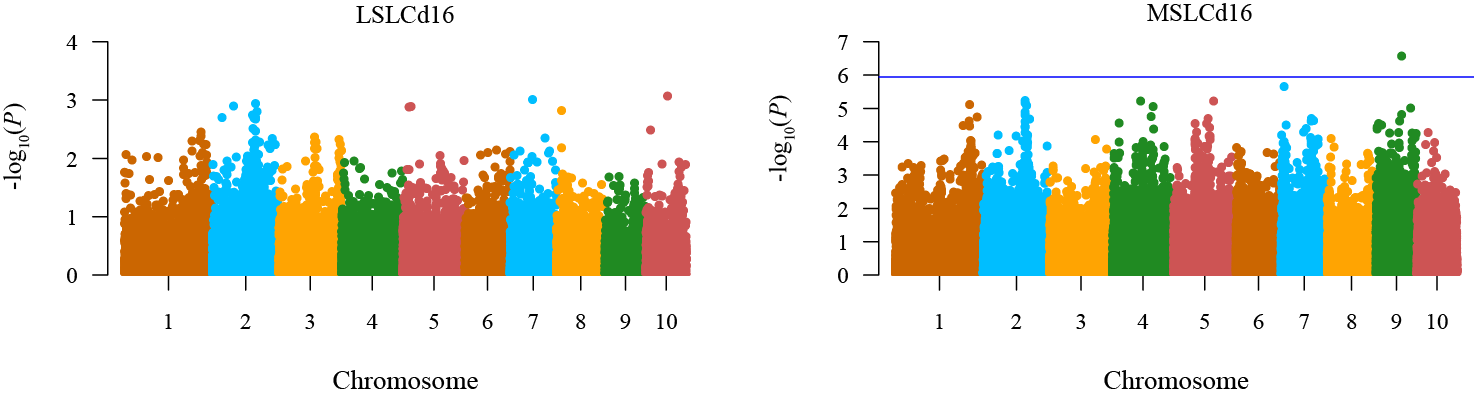

Supplement: Supplementary file 4 — Manhattan plots of association analysis for leaf Cd concentration at seeding stage of maize in 2016. (PNG 64 kb) [file 12864_2017_4395_MOESM4_ESM.png]

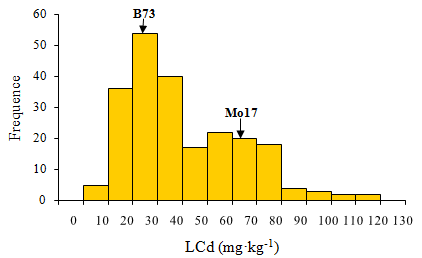

Supplement: Supplementary file 6 — The frequency distribution of leaf Cd concentration in maize IBMSyn10 double haploid (DH) population. (PNG 7 kb) [file 12864_2017_4395_MOESM6_ESM.png]
